# Case Report: Balancing immunosuppression and infection—intravenous immunoglobulin in anti-HMGCR immune-mediated necrotizing myopathy complicated by severe pneumonia

**DOI:** 10.3389/fmed.2026.1920824

**Published:** 2026-08-26

**Authors:** Jing Fu, Qiong Zhao, Jing-Bo Lou, Gui-Hua Tang, Wei Chen, Jiang-Hu Zhao, Qian-Song He

**Affiliations:** 1 First Clinical Medical College, Guizhou University of Traditional Chinese Medicine, Guiyang, China; 2 The Second Affiliated Hospital of Guizhou University of Traditional Chinese Medicine, Guiyang, China; 3 The First Affiliated Hospital of Guizhou University of Traditional Chinese Medicine, Guiyang, China

**Keywords:** anti-HMGCR antibodies, immune-mediated necrotizing myopathy, intravenous immunoglobulin, respiratory failure, sepsis, statins

## Abstract

A 65-year-old male with a 3-year history of atorvastatin use presented with progressive proximal muscle weakness, dysphagia, and markedly elevated creatine kinase (2,277 U/L). Initially misdiagnosed with polymyositis, he deteriorated with severe pneumonia, septicemia, and type I respiratory failure requiring mechanical ventilation. Interleukin-6 rose from 12.83 to 610.9 pg/mL during sepsis. After confirmation of anti-HMGCR antibody positivity (18 arbitrary units [AU], cutoff >10 AU) via line blot assay, we discontinued methotrexate, maintained methylprednisolone at 40 mg, and initiated intravenous immunoglobulin (IVIG) 0.4 g/kg/day for 5 days with broad-spectrum antibiotics. Within two weeks, proximal muscle strength (MRC scale) improved from 2/5 to 4/5, IL-6 normalized to 8.56 pg/mL, and respiratory failure resolved. This case suggests that IVIG may serve as a safe immunomodulatory bridge in critically ill IMNM patients with severe infections.

## Introduction

Statins are widely used for primary and secondary prevention of atherosclerotic cardiovascular disease. While generally well-tolerated, they can cause muscle-related adverse events ranging from mild myalgia to severe immune-mediated necrotizing myopathy (IMNM) (1, 2). Anti-HMGCR antibody-positive IMNM is a distinct subtype strongly associated with prolonged statin exposure. While lipophilic statins such as atorvastatin are the most frequently implicated triggers, hydrophilic statins such as rosuvastatin have also been reported to induce this condition (3). The condition is characterized by severe proximal weakness, markedly elevated creatine kinase (CK), and muscle biopsy showing necrosis with regeneration but minimal inflammatory infiltration (4).

The management of anti-HMGCR IMNM becomes extremely challenging when complicated by severe infections. Conventional immunosuppressive therapies—particularly high-dose corticosteroids—can exacerbate sepsis and respiratory failure in critically ill patients. Intravenous immunoglobulin (IVIG) has emerged as a potential steroid-sparing agent with a favorable safety profile (5), but evidence supporting its use in IMNM patients with concurrent life-threatening infections remains limited.

Here we report a case of anti-HMGCR IMNM complicated by severe pneumonia and respiratory failure, in which IVIG facilitated a successful outcome. We also provide a focused literature review of 25 published cases to support this therapeutic strategy (6–29).

## Case description

A 65-year-old male with a history of hypertension, cerebral infarction, and hyperlipidemia, along with a 40-year history of smoking and alcohol consumption, had been taking atorvastatin 20 mg daily for three years.

**Figure 1:**
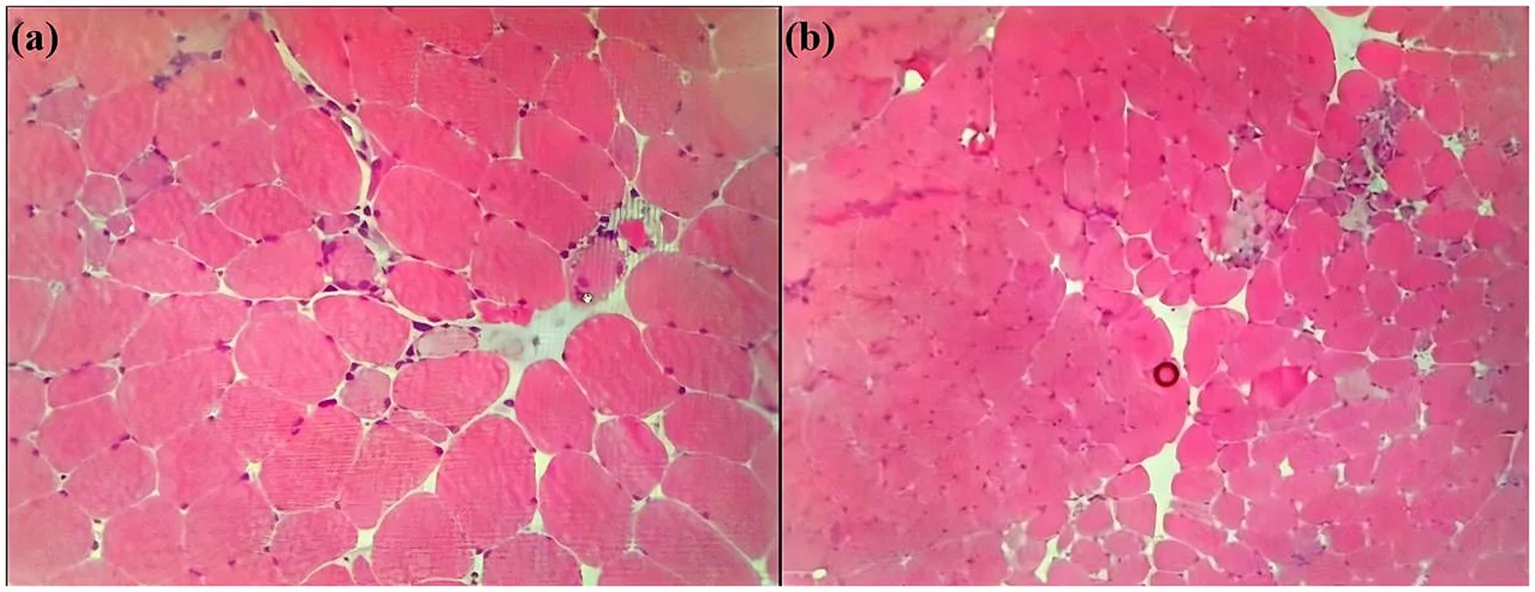
Thigh muscle biopsy (Hematoxylin and eosin staining). **(a,b)** Show diffuse muscle fiber necrosis with regenerative changes (basophilic fibers with large nuclei). Notably, there is an absence of prominent inflammatory cell infiltration, which is characteristic of IMNM rather than polymyositis.

*Initial presentation (two months prior to admission)*: The patient developed progressive weakness in all four limbs, inability to lift his neck, and difficulty swallowing. At that time, MRC scale strength was: neck 3/5, proximal upper limbs 3/5, distal upper limbs 4/5, proximal lower limbs 3/5, and distal lower limbs 4/5. He visited a local hospital where a thigh muscle biopsy (Figure 1) showed diffuse muscle fiber necrosis with regenerative changes but scant inflammatory infiltration. He was diagnosed with polymyositis and treated with oral methotrexate (10 mg weekly), oral methylprednisolone (40 mg daily), and tripterygium wilfordii (TWP), with no significant improvement.

*Admission to our hospital (Day 1)*: Two weeks later, his weakness worsened severely; he was unable to walk or lift his arms. On admission, he presented with shortness of breath, chest tightness, productive cough, slurred speech, and dysphagia. Physical examination revealed marked proximal muscle atrophy. Neck muscle strength was graded 2/5, proximal upper limb strength 3/5 (distal 4/5), and proximal lower limb strength 2/5 (distal 4/5) using the MRC scale. Initial laboratory tests showed CK 2,277 U/L (normal <190), AST 174 U/L, ALT 207 U/L, and interleukin-6 (IL-6) 12.83 pg/mL (Table 1).

**Table 1:** Dynamic changes in key laboratory parameters during hospitalization.

Parameter (normal range)	Day 1	Day 7	Day 10 (IVIG initiation + MTX stopped)	Day 14	Day 18
Intervention	Admission	Deterioration	IVIG 25 g/d started; MTX discontinued	Ongoing	Recovery
CK (0–190 U/L)	2,277	–	2,184	–	897
IL-6 (0–7 pg/mL)	12.83	48.24	610.9	13.88	8.56
CRP (0–10 mg/L)	24.8	11.35	14.55	74.62	8.0
WBC (3.5–9.5 × 10^9^/L)	10.65	12.06	10.97	10.26	8.6
ALT (0–42 U/L)	207	198	228	174	156
AST (0–42 U/L)	174	114	219	151	137

IVIG was initiated and methotrexate was discontinued on Day 10. The marked decline in CK and IL-6 by Day 18 indicates a favorable response to immunotherapy without exacerbating infection. CRP, C-reactive protein; WBC, white blood cell count; ALT, alanine aminotransferase; AST, aspartate aminotransferase.

*Clinical deterioration (Day 7–10)*: Despite continued treatment for polymyositis, the patient developed high fever (38.3 °C), worsening dyspnea, and type I respiratory failure (PaO₂ 50 mmHg, PCO₂ 34.8 mmHg). Inflammatory markers surged: IL-6 rose to 610.9 pg/mL, white blood cell count to 12.3 × 10^9^/L, and C-reactive protein to 33.78 mg/L. Chest CT revealed multiple scattered inflammatory infiltrates in both lungs (Supplementary Figure 1). Blood cultures confirmed septicemia. Mechanical ventilation was initiated. Of note, the Day 7 CK value was not available as the patient was transferred emergently from the referring hospital. The modest CK change from Day 1 (2,277 U/L) to Day 10 (2,184 U/L) despite marked clinical deterioration likely reflects the well-recognized dissociation between CK levels and clinical severity in chronic anti-HMGCR IMNM, particularly in the setting of advanced muscle atrophy, where CK may plateau or decline slowly even with active disease.

This life-threatening infection posed a critical therapeutic dilemma: escalating immunosuppression risked overwhelming sepsis, but withholding it risked irreversible myopathy.

*Therapeutic intervention and response (Day 10–18)*: After obtaining myositis-specific antibody results (using a line blot assay [Euroimmun, Germany], with results expressed in arbitrary units [AU] based on a semi-quantitative readout) (Table 2), which confirmed positivity for anti-HMGCR IgG (18 AU, normal <5, cutoff for positive >10 AU) and anti-SSA/Ro52 (21 AU), we made a deliberate decision to discontinue methotrexate immediately to avoid further myelosuppression and immune impairment in the setting of bacteremia. We maintained methylprednisolone at 40 mg daily without dose escalation, and initiated IVIG at 25 g (0.4 g/kg) daily for 5 consecutive days, alongside broad-spectrum antibiotics and ventilatory support. Prior to IVIG administration, serum IgA level was measured (2.15 g/L, normal range 0.7–4.0 g/L), ruling out IgA deficiency and mitigating anaphylaxis risk. Renal function was closely monitored (baseline creatinine 82 μmol/L, peak 96 μmol/L during IVIG therapy), and the patient received adequate hydration to minimize the risk of IVIG-associated nephrotoxicity. Thromboembolic risk was assessed as low based on the patient’s ambulatory status, absence of prior thrombotic events, and normal coagulation parameters, and no prophylactic anticoagulation was administered.

**Table 2:** Semi-quantitative detection of myositis autoantibody profile.

Antibody	Result (AU)	Reference interval	Interpretation
Anti-HMGCR IgG	18.0	Negative <5; Positive >10	Positive
Anti-SSA/Ro52 IgG	21.0	Negative <5; Positive >10	Positive
Anti-Jo-1, PL-7, PL-12, EJ, OJ, SRP, Mi-2, MDA-5, TIF-1γ, SAE-1/2, etc.	<5.0	Negative	Negative

Detection method was line blot assay (Euroimmun, Germany). Only anti-HMGCR and anti-SSA/Ro52 antibodies were positive, confirming the diagnosis of anti-HMGCR IMNM.

**Figure 2:**
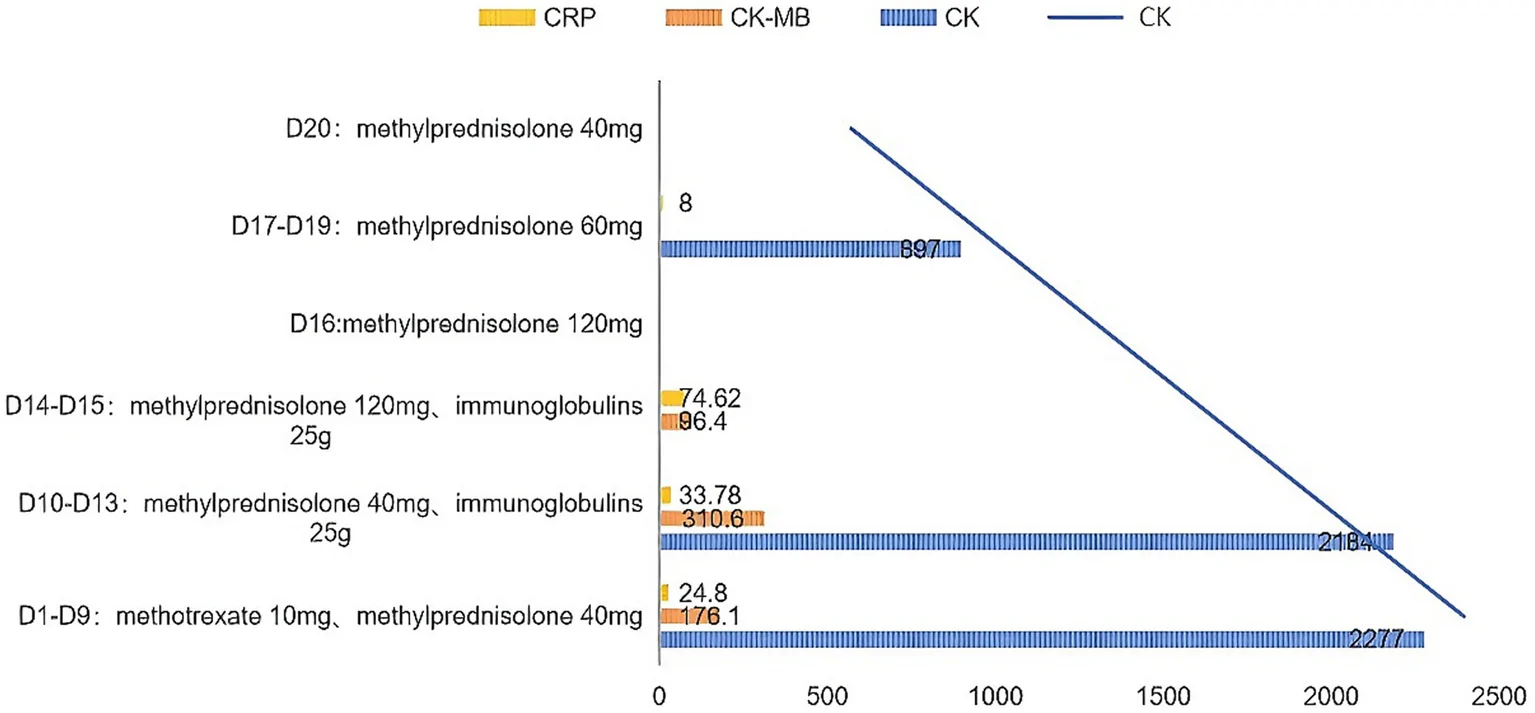
Timeline of clinical events and biomarker trends during hospitalization. CK, creatine kinase; IL-6, interleukin-6; CRP, C-reactive protein; WBC, white blood cell count; IVIG, intravenous immunoglobulin; MTX, methotrexate; ABx, antibiotics; Vent, ventilation. The graph illustrates the sharp rise in IL-6 corresponding to severe pneumonia (Day 10), followed by a rapid decline in both IL-6 and CK after the initiation of IVIG and discontinuation of methotrexate, demonstrating the efficacy of this approach.

The patient’s response was striking. Within 72 h, fever subsided and respiratory parameters improved. By Day 18, IL-6 dropped to 8.56 pg/mL, white blood cell count normalized to 8.6 × 10^9^/L, and CK decreased to 897 U/L (Figure 2; Table 1). Neurological examination at discharge showed significant improvement: neck strength 4/5, proximal upper limbs 4/5 (distal 5/5), and proximal lower limbs 3/5 (distal 5/5). Swallowing and speech intelligibility were markedly restored. The patient was discharged with instructions to discontinue atorvastatin permanently and continue oral methylprednisolone 40 mg with a tapering schedule: reduce by 5 mg every 2 weeks until 20 mg daily is reached, then reduce by 2.5 mg every 2 weeks until discontinuation, with close clinical monitoring for relapse. The patient was also prescribed trimethoprim-sulfamethoxazole (one single-strength tablet daily) for Pneumocystis jirovecii pneumonia prophylaxis, and oral calcium carbonate (500 mg twice daily) with vitamin D3 (800 IU daily) for bone protection, in accordance with rheumatological guidelines for patients on long-term glucocorticoid therapy. Regular follow-up was advised.

At 90-day follow-up, the patient remained clinically stable. His MRC strength had further improved: neck 5/5, proximal upper limbs 5/5, distal upper limbs 5/5, proximal lower limbs 4/5, and distal lower limbs 5/5. He was ambulating independently with a cane. CK had declined to 186 U/L, and IL-6 was 3.2 pg/mL. Methylprednisolone had been tapered to 15 mg daily without evidence of relapse. No new infectious complications had occurred.

## Discussion

To contextualize our management strategy, we systematically reviewed the literature on statin-associated anti-HMGCR IMNM published up to 2025, identifying 25 eligible case reports and series (6–29). The pooled data (Supplementary Table 1) confirmed our patient’s typical profile: prolonged statin exposure (mean 2.8 years, 92% of cases), with atorvastatin being the most common (68%). The hallmark features were severe hyperCKemia (mean 11,237 U/L), proximal weakness (mean MRC score 2.8/5), and dysphagia (34.6%). Critically, severe respiratory complications requiring ventilation were reported in only 15.4% of cases, underscoring the rarity and critical nature of our patient’s condition.

The initial misdiagnosis as “polymyositis” is a recurring theme in the literature, largely because the distinct nosology of IMNM was only established in 2003 (1, 4). This case reinforces the necessity of early myositis-specific antibody testing in any patient with unexplained proximal weakness and a history of statin use, especially before initiating aggressive immunosuppression. The anti-HMGCR titer of 18 AU, while clearly positive per the manufacturer’s cutoff (>10 AU), is relatively low compared to titers often reported in classic anti-HMGCR IMNM (100 s–1,000s AU). However, the presence of a positive anti-HMGCR antibody in the appropriate clinical context—proximal weakness, marked CK elevation, and characteristic muscle biopsy findings—is diagnostically confirmatory regardless of titer. The concurrent anti-SSA/Ro52 positivity (21 AU) raises the possibility of an overlap autoimmune phenotype, as anti-SSA/Ro52 antibodies are frequently observed in various connective tissue diseases and may be associated with more severe myopathy or extramuscular manifestations. Nonetheless, the clinical features and biopsy findings in this case were most consistent with anti-HMGCR IMNM, and the presence of anti-SSA/Ro52 did not alter our management approach.

The core clinical lesson from this case lies in the therapeutic decision-making. In patients with active, severe infection (IL-6 > 600 pg/mL, septicemia), conventional high-dose pulse corticosteroids or rituximab carry substantial risks of immunosuppression and pathogen reactivation (5, 30). Moreover, continuation of methotrexate in this setting would have compounded the risk of bone marrow suppression and delayed infection clearance. Therefore, we elected to stop methotrexate and avoid escalating corticosteroids, relying instead on IVIG as the primary immunomodulatory agent. The improvement observed in this case cannot be definitively attributed to IVIG alone, as three interventions (IVIG initiation, methotrexate discontinuation, and broad-spectrum antibiotics/ventilatory support) were initiated concurrently. It is likely that the favorable outcome resulted from a synergistic effect of infection control and immunomodulation. Nevertheless, IVIG’s established mechanisms—including neutralization of pathogenic autoantibodies, Fc receptor modulation, and attenuation of cytokine storms—provide a biologically plausible rationale for its use in this setting, without the bone marrow suppression associated with conventional immunosuppressants.

To further contextualize our therapeutic approach, we review the broader evidence for IVIG and other rescue therapies in idiopathic inflammatory myopathies (IIM). A recently updated Cochrane systematic review (2025) evaluated non-targeted immunomodulatory therapies in IIM and found moderate-certainty evidence that IVIG improves disability, muscle strength, and skin manifestations in dermatomyositis, though evidence for other IIM subtypes, including IMNM, remains limited due to a paucity of randomized controlled trials (31). The application of IVIG in rare autoimmune diseases parallels its use in systemic sclerosis (SSc), where a large multicenter study demonstrated benefit across multiple organ systems—including musculoskeletal, gastrointestinal, and cutaneous manifestations—with an acceptable safety profile (32). This analogy supports the rationale for IVIG use in severe, treatment-refractory IMNM where conventional immunosuppression carries prohibitive infection risks.

For patients who fail to respond to IVIG or require additional disease control, rituximab (RTX) represents an important second-line rescue option. The ENMC has recommended RTX as a second-line therapeutic option for IMNM based on accumulating case series and real-world evidence (3, 26). A study of refractory IIM patients demonstrated that RTX treatment was associated with significant steroid-sparing effects, with favorable treatment persistence and remission rates (33). Importantly, RTX tapering (e.g., 1 g every 6 months) proved safe and effective in maintaining remission in eligible patients, with no disease flares or adverse drug reactions reported among those tapered, suggesting that dose reduction strategies can improve treatment tolerability without compromising efficacy (34).

Emerging evidence also informs glucocorticoid-sparing strategies in anti-HMGCR IMNM. A multicentric study of 24 anti-HMGCR-positive patients found that a glucocorticoid-free regimen based on IVIG achieved remission in all 5 patients who received it, with similar time to remission compared to standard glucocorticoid-containing protocols (35). This observation aligns with our management strategy of maintaining rather than escalating methylprednisolone during active infection, suggesting that judicious corticosteroid use may optimize outcomes while minimizing immunosuppressive complications.

In our reviewed cohorts (Supplementary Table 2), IVIG was used in 56% of studies, predominantly for refractory or severe cases, and was associated with rapid strength improvement in 85.7% of recipients (6–29). Our patient’s recovery of proximal strength from 2/5 to 4/5 within two weeks aligns with this data.

The transient elevation of C-reactive protein observed on Day 14 (74.62 mg/L) merits comment. To rule out a secondary nosocomial infection, repeat blood cultures and sputum cultures were obtained on Day 14, both of which were negative. The patient remained afebrile with improving oxygenation, supporting the interpretation of the CRP elevation as an IVIG-associated acute-phase reaction rather than a new infectious process.

The relatively modest CRP elevation (33.78 mg/L) at the time of the IL-6 surge (610.9 pg/mL) merits comment. While this dissociation is atypical for classic bacterial sepsis, it may reflect the concurrent immunosuppressive effect of the patient’s ongoing methylprednisolone therapy (40 mg daily), which is known to attenuate the hepatic CRP response to IL-6. Alternatively, this pattern may indicate a significant contribution from the underlying autoimmune inflammatory process to the IL-6 elevation, rather than infection alone.

*Study limitations*: This is a single case report, and the findings may not be generalizable. The patient did not undergo genetic screening for statin-related polymorphisms (e.g., HLA-DRB1*11:01, SLCO1B1) due to financial constraints (3). We have provided 90-day follow-up data, but longer-term outcomes remain unknown. Additionally, the temporal relationship between IVIG initiation and clinical improvement, while compelling, does not prove causality, and the concurrent initiation of multiple interventions prevents attribution of benefit to IVIG alone.

*Take-home messages*: In statin-associated anti-HMGCR IMNM complicated by severe pneumonia and respiratory failure, IVIG may represent a safe and effective therapeutic option. It allows for the control of myopathy without exacerbating systemic infection, especially when conventional immunosuppressants like methotrexate are withdrawn and corticosteroids are not escalated. We suggest that clinicians consider early IVIG in similar high-risk scenarios, combined with aggressive infection management, to break the vicious cycle between autoimmunity and sepsis. A multidisciplinary approach involving neurologists, intensivists, and infectious disease specialists is paramount for optimal outcomes.

## Patient perspective

The patient and his family provided the following perspective on his treatment experience:

“When my father became unable to walk, lift his arms, or even swallow food, our family was extremely worried. The doctors explained that he had a rare immune reaction to his cholesterol-lowering medication, which was further complicated by a severe lung infection. We were told that conventional treatments might worsen his infection, which frightened us. When the medical team proposed IVIG as an alternative, we understood that this treatment could help control the muscle damage without further suppressing his ability to fight the infection. Within days of starting IVIG, we began to see improvement—he could speak more clearly, swallow with less difficulty, and gradually move his arms and legs again. By the time he was discharged, he could stand with assistance. We are grateful for the careful and individualized approach taken by the medical team, who always kept us informed and addressed our concerns throughout this difficult journey.”

## Data Availability

The original contributions presented in the study are included in the article/Supplementary material, further inquiries can be directed to the corresponding authors.

## Supplementary material

SUPPLEMENTARY FIGURE 1. Chest CT: multiple scattered inflammatory infiltrates in both lungs, consistent with severe pneumonia.
