# Supplementary material for: Case Report: Balancing immunosuppression and infection—intravenous immunoglobulin in anti-HMGCR immune-mediated necrotizing myopathy complicated by severe pneumonia
Source: Front Med (Lausanne). 2026 Aug 26;13:1920824. doi: 10.3389/fmed.2026.1920824 (PMC13553862; doi:10.3389/fmed.2026.1920824)
Supplement: Supplementary file 1 [file Table_2.DOCX]

**Supplementary table 2.** Summary of Treatment Strategies and Efficacy for Anti-HMGCR Antibody-Positive IMNM (Based on Analysis of 25 Studies)

| **Intervention** | **Category** | **Representative Regimen** | **Clinical Efficacy** | **Remarks** |
| --- | --- | --- | --- | --- |
| Corticosteroids | First-line | Prednisone 1 mg/kg/day; Methylprednisolone pulse (1 g/day × 3–5 days) | Good initial response, but prone to relapse when used alone; requires combination therapy | Requires slow tapering; monitor for osteoporosis, infection, etc. |
| Conventional Immunosuppressants | First-line (Adjunct) | Methotrexate 10–25 mg/week; Azathioprine 1.5–2 mg/kg/day; Mycophenolate mofetil 1–3 g/day | Effective as steroid-sparing agents; relatively slow onset (1–3 months) | Monitor liver/kidney function, bone marrow suppression; methotrexate most commonly used |
| **Intravenous Immunoglobulin (IVIG)** | **Second-line / Rescue** | **2 g/kg divided over 2–5 days; 0.4 g/kg/day × 5 days** | **Rapid improvement in muscle strength (within weeks), especially effective in severe, refractory cases and patients with concurrent infection** | **Favorable safety profile; suitable for patients with concurrent infection (as in this case)** |
| Rituximab | Second-line / Rescue | 375 mg/m² weekly × 4 or 1 g × 2 doses (2 weeks apart) | Effective in cases refractory to conventional therapy and IVIG | May increase infection risk; expensive; requires B-cell monitoring |
| Therapeutic Plasma Exchange (TPE) | Adjunctive | 3–5 sessions per week | Short-term adjunct in acute phase; effects not sustained | Requires combination with immunosuppressive therapy; resource-intensive |
