# Supplementary material for: Case Report: Balancing immunosuppression and infection—intravenous immunoglobulin in anti-HMGCR immune-mediated necrotizing myopathy complicated by severe pneumonia
Source: Front Med (Lausanne). 2026 Aug 26;13:1920824. doi: 10.3389/fmed.2026.1920824 (PMC13553862; doi:10.3389/fmed.2026.1920824)
Supplement: Supplementary file 2 [file Table_1.DOC]

Supplementary table 1. Summary of Basic Characteristics of Included Studies (n=25)

|  | **Characteristic** | **Value/Proportion** | **Remarks** |
| --- | --- | --- | --- |
| **Study Design** | Case Reports | 22 (88%) |  |
|  | Systematic Reviews | 3 (12%) |  |
|  | Total Patients | 26 | One review included 3 cases; others were single cases |
| **Demographics** | Mean Age | 64.2 years | Range: 43–87 years |
|  | Sex Ratio (M:F) | 13:13 | 1:1 |
| **Statin Exposure** | Statin-associated | 23 (92%) |  |
|  | Mean Duration of Use | 2.8 years | Range: 2 months–7 years |
|  | Predominant Statin Type | Atorvastatin (68%) |  |
| **Clinical Presentation** | Mean Peak CK Level | 11,237 U/L | Range: Normal–40,476 U/L |
|  | Mean Muscle Strength Grade | 2.8/5 | Range: 1–4/5 |
|  | Dysphagia | 9 cases (34.6%) |  |
|  | Respiratory Complications | 4 cases (15.4%) |  |
| **Diagnostic Methods** | Muscle Biopsy | 22 (88%) |  |
|  | Anti-HMGCR Antibody Test | 25 (100%) |  |
|  | Imaging (MRI) | 10 (40%) |  |
| **Treatment Strategies** | Corticosteroids | 25 (100%) |  |
|  | Conventional Immunosuppressants | 21 (84%) |  |
|  | IVIG Therapy | 14 (56%) |  |
|  | Rituximab | 7 (28%) |  |
